# Supplementary figures and images for: Integrating sex-bias into studies of archaic introgression on chromosome X
Source: PLoS Genet. 2023 Aug 14;19(8):e1010399. doi: 10.1371/journal.pgen.1010399 (PMC10449224; doi:10.1371/journal.pgen.1010399)

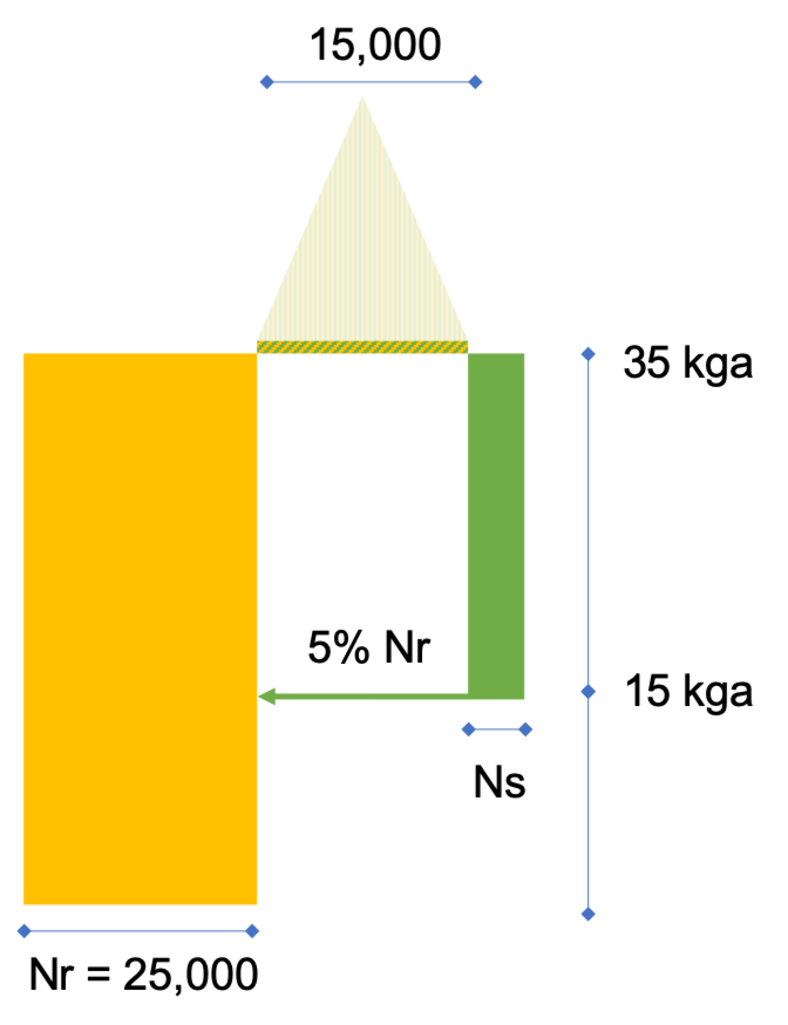

Supplement: S1 Fig — Nr indicates size of recipient population in haplotypes; Ns indicates size of source population in haplotypes; kga indicates thousand generations ago. Results presented here have Nr = 100Ns. Filled colors indicate generations in forward simulation; a prior coalescent history is appended to the ancestral population. See Methods for further details. (TIF) [file pgen.1010399.s001.tif]

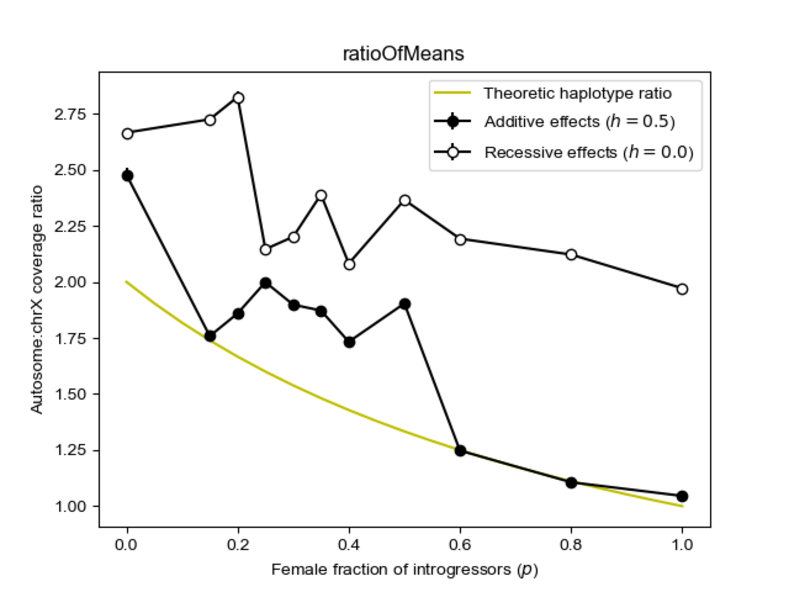

Supplement: S2 Fig — Chartreuse line depicts autosome to chromosome X haplotype ratio of a theoretical population with a given male fraction (Eq 1). Error bars are bootstrapped 95% confidence intervals around the ratio of 10,000 means generated from autosomal and chromosome X coverage distributions; in most cases error bars are smaller than the displayed points. (TIF) [file pgen.1010399.s002.tif]

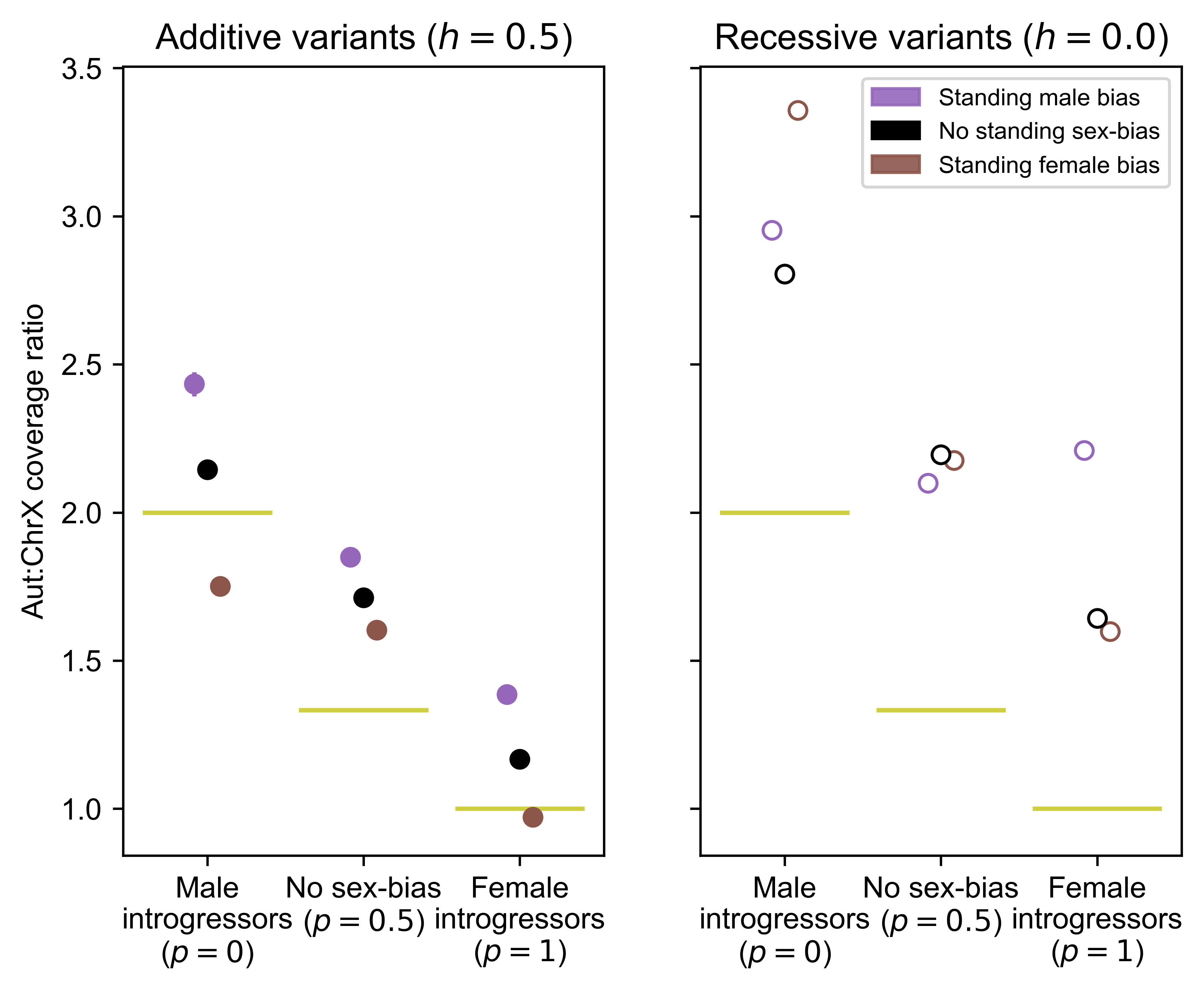

Supplement: S3 Fig — A Aut:ChrX coverage ratios from simulations that reflect both sex-bias within the introgression pulse, and a constant, (“standing”) unequal sex ratio in the recipient population. Sex ratios shown are 25% female (standing male bias), 50% female (no standing sex-bias), and 75% female (standing female bias). Black points reflecting no standing sex-bias are equivalent to those shown in Fig 3A and 3C. Error bars are bootstrapped 95% confidence intervals around the ratio of 10,000 means generated from autosomal and chromosome X coverage distributions; in most cases the error bars are smaller than the displayed points. All variants are additive (h = 0.5). B Same as panel A, but all variants are recessive (h = 0). (TIF) [file pgen.1010399.s003.tif]

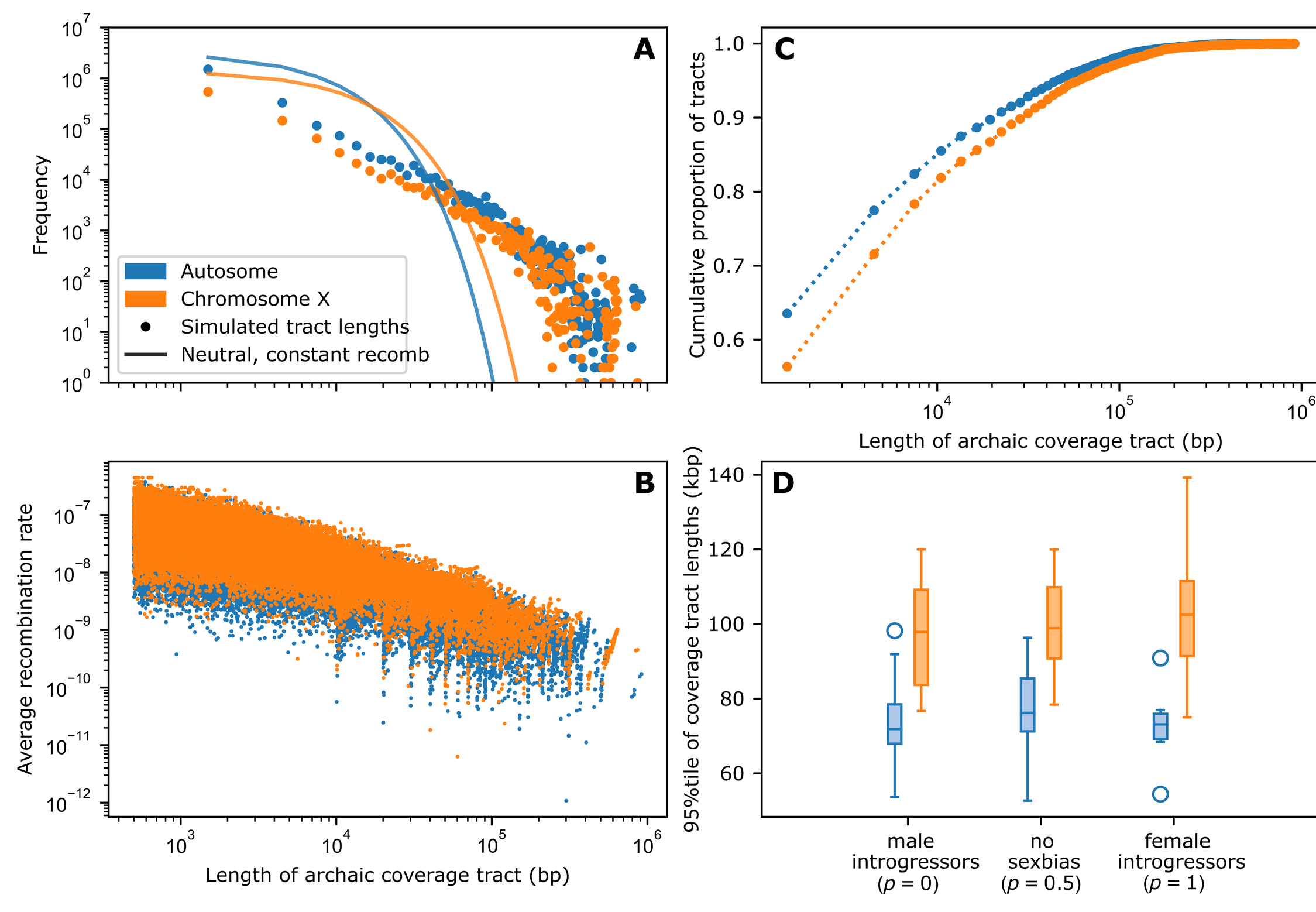

Supplement: S4 Fig — All results shown reflect an additive model of dominance. A Tract length spectra from simulations of an archaic introgression scenario under autosomal or chromosome X inheritance; note log scale. Points indicate frequencies of archaic coverage tracts within length bins of 3000 bp. Simulated chromosomes shared a variable recombination rate landscape (see Methods for details). Solid lines indicate expected archaic coverage lengths under a simple, neutral model with constant recombination rate equal to the mean rate of the simulated recombination landscape. B Average recombination rate within each simulated archaic coverage tract, plotted against length of the tract. Note log scale. C Proportion of archaic coverage tracts that are a given length or shorter; proportions are calculated within each inheritance type. Arrow indicates 95th percentile of tract lengths, illustrated in panel D alongside sex-biased scenarios. D Distribution of 95th percentiles of archaic coverage tract lengths found on either an autosome or chromosome X, across degrees of introgressor sex-bias. Each box reflects the length distributions from ten simulation replicates. (TIF) [file pgen.1010399.s004.tif]

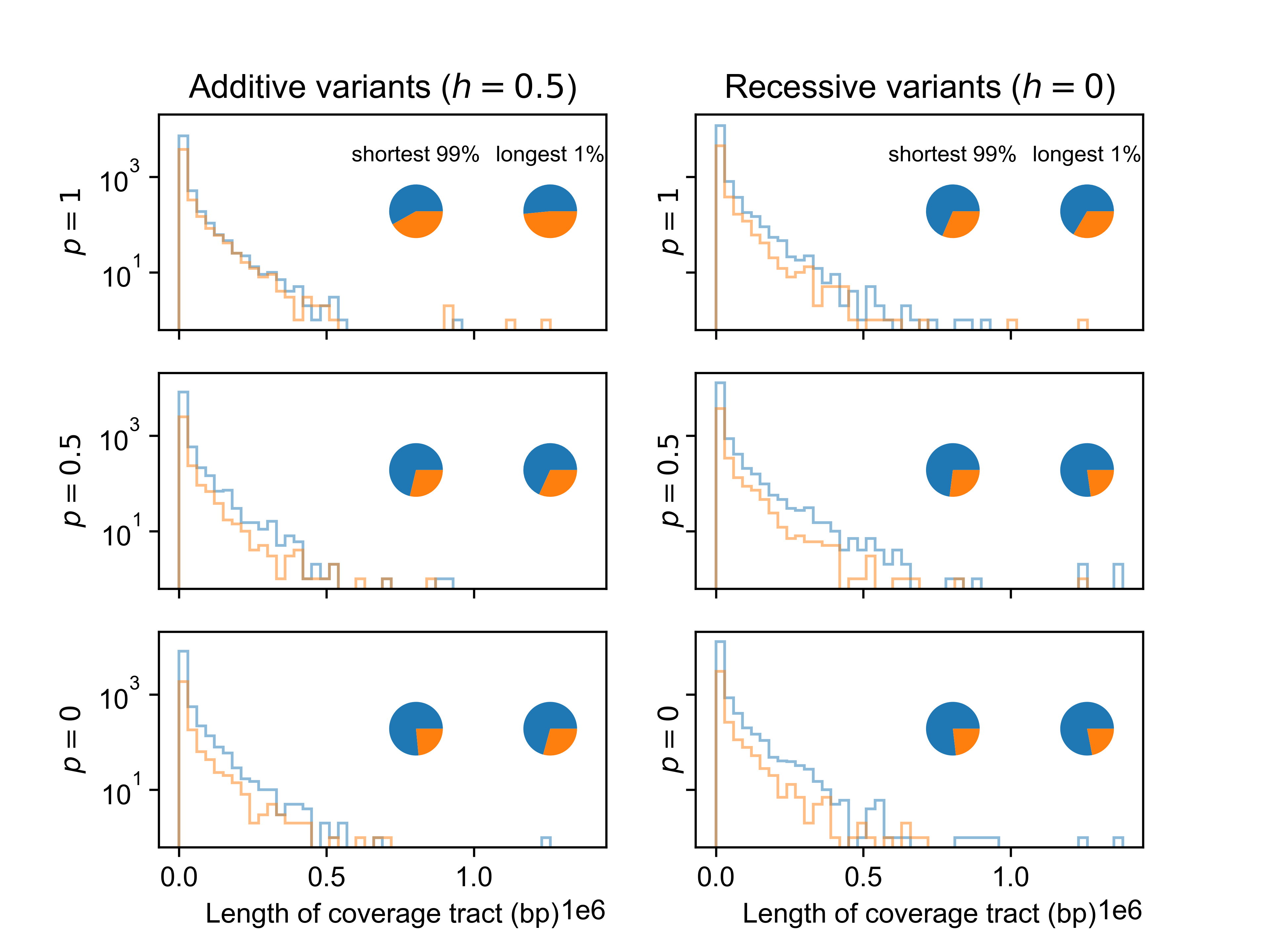

Supplement: S5 Fig — Archaic coverage tract length spectra from a simulated autosome (blue) and chromosome X (orange). Each plot represents a combination of dominance (h = 0 or h = 0.5) and sex-bias (p = 0, p = 0.5, or p = 1, where p is the female fraction of the introgressors). Pie charts depict fraction of total coverage found on the autosome or chromosome X. Right pie shows coverage contained within the longest 1% of tracts; left pie shows all other coverage. Note that chromosomes have identical size and local recombination rates; see Methods. (TIF) [file pgen.1010399.s005.tif]

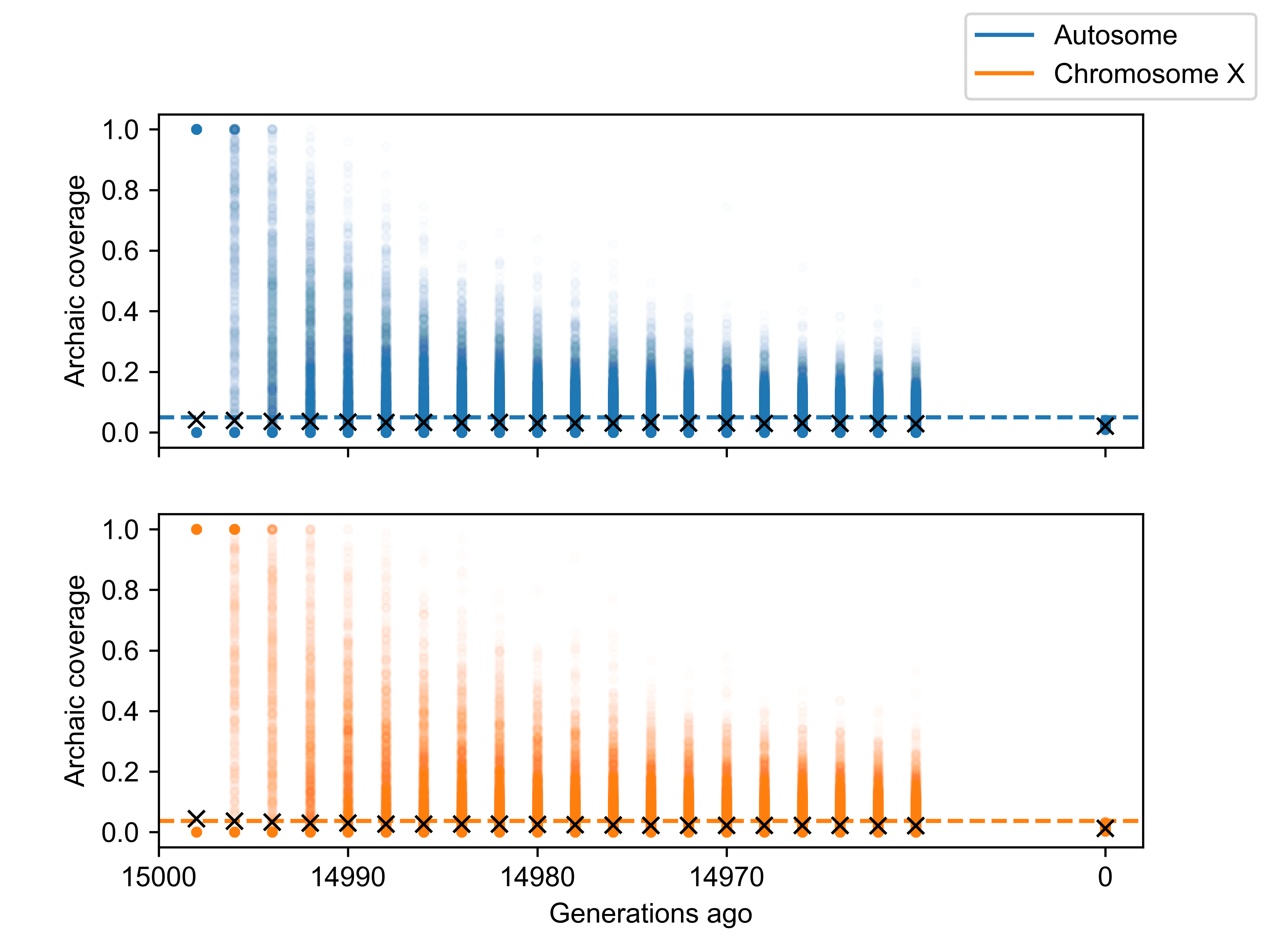

Supplement: S6 Fig — Each point (note transparency) reflects the archaic coverage on one of 1000 haplotypes sampled from the recipient population every two generations for the first 40 generations after the introgression event. Crosses indicate mean coverage at each timepoint (see Fig 4). Horizontal line indicates initial introgression fraction. (TIF) [file pgen.1010399.s006.tif]

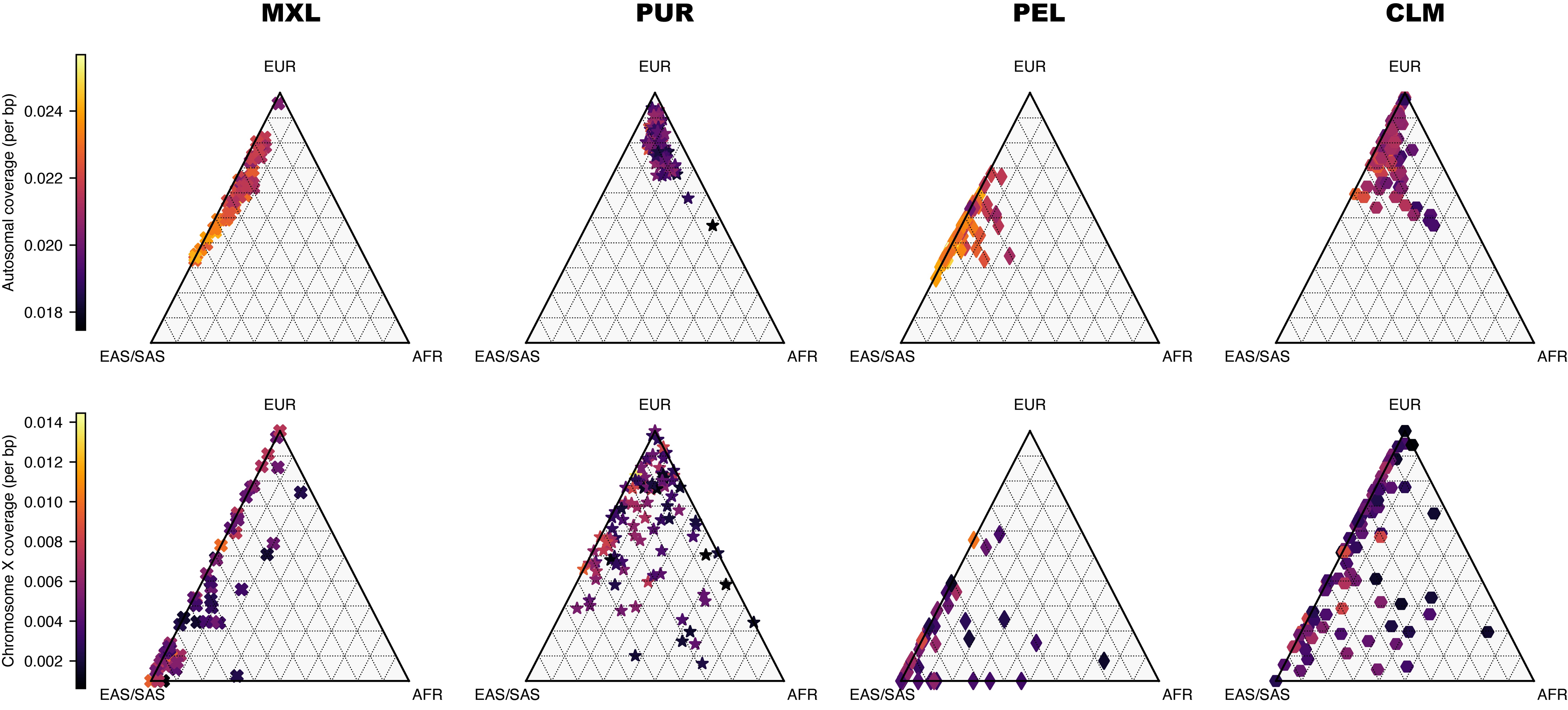

Supplement: S7 Fig — Colors indicate per-base pair archaic coverage estimates on autosomes or chromosome X, inferred by method of Skov et al. [5] using data from AMR samples from the 1000 Genomes Project [18], selected as described in Methods. Coordinates are ADMIXTURE [25] ancestry estimates for the individuals, using supervised clustering with K = 3 and EUR, AFR, and combined EAS and SAS individuals as reference groups. (TIF) [file pgen.1010399.s007.tif]

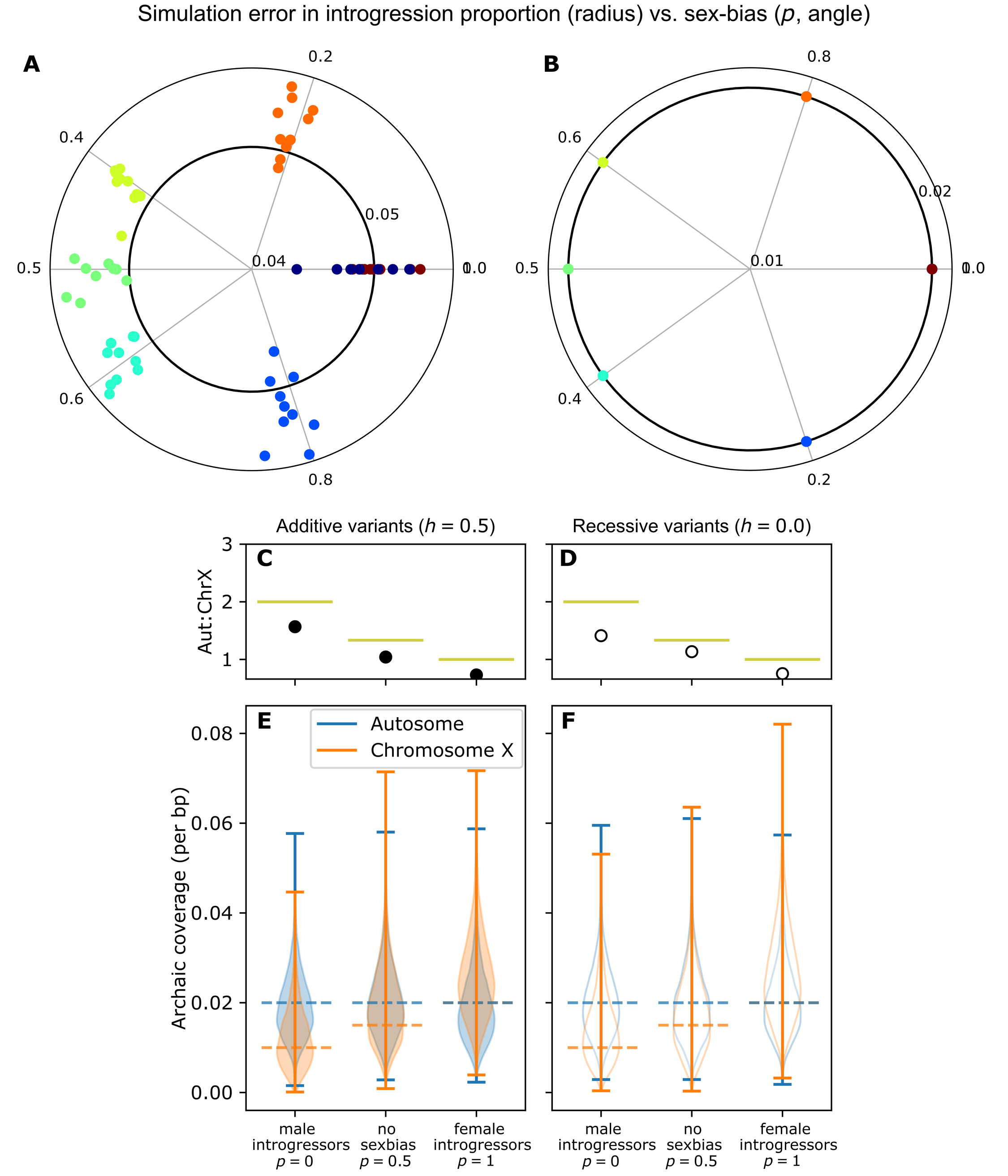

Supplement: S8 Fig — A Our simulations contain some stochasticity in both introgression proportion and introgressor sex ratio (p). Each point is an independent simulation run of the demographic model. The radius indicates the number of introgressing individuals as a proportion of recipient population (0.05 was simulated). The angle indicates the fraction of introgressors that were female (p = 0, 0.2, 0.4, 0.5, 0.6, 0.8, 1 were simulated). The points plotted here for p = 0, 0.5, 1 are the same simulations as those in Figs 3A, 3C and 4B. B Same as panel A, but from ten simulations at each p using a revised simulation approach with no stochasticity in introgression proportion or sex-bias. Note that ten replicate points are all plotted at the same location. (Introgression fraction of 0.02 was simulated, rather than 0.05 as in panel A and the rest of the paper.) Panels C-F are equivalent to Fig 3, but use data from panel B. The mean ratios in panels C and D in this “neutral” scenario systematically underestimate the theoretical haplotype ratio expected for each sex-bias scenario. (TIF) [file pgen.1010399.s008.tif]
