# Supplementary material for: Integrating sex-bias into studies of archaic introgression on chromosome X
Source: PLoS Genet. 2023 Aug 14;19(8):e1010399. doi: 10.1371/journal.pgen.1010399 (PMC10449224; doi:10.1371/journal.pgen.1010399)
Supplement: S1 Table — Coverage is the per-base pair proportion of the genome inferred to have come from archaic sources, found either on the combined 22 autosomes, or on chromosome X. The standard deviation across individuals is in parentheses. The fourth column shows the ratio of autosomal archaic coverage to chromosome X archaic coverage (Aut:chrX), which is always at least equal, and can range up to 18.8. All groups outside of Continental Africa have at least 3.3 times more relative coverage on autosomes than chromosome X. (PDF) [file pgen.1010399.s009.pdf]

**Method** *description*	**Autosome coverage**	**ChrX coverage**	**Aut:ChrX ratio**	**Continental group**	**Group** *data source*	**Archaic reference**	**Citation**
Sankararaman *et al.*, 2014							
Conditional Random Field					*1k Genomes*	Altai	[1]
	1.20% (0.07)	0.19% (0.14)	6.3	EUR	FIN		
	1.22% (0.09)	0.21% (0.15)	5.8	AMR	MXL		
	1.15% (0.08)	0.20% (0.15)	5.8	EUR	GBR		
	1.17% (0.08)	0.21% (0.17)	5.6	EUR	CEU		
	1.38% (0.10)	0.26% (0.21)	5.3	EAS	JPT		
	1.05% (0.12)	0.20% (0.15)	5.3	AMR	PUR		
	1.14% (0.12)	0.22% (0.16)	5.2	AMR	CLM		
	1.37% (0.08)	0.27% (0.21)	5.1	EAS	CHS		
	0.34% (0.22)	0.07% (0.11)	4.9	AFR	ASW		
	1.40% (0.08)	0.30% (0.21)	4.7	EAS	CHB		
	1.07% (0.06)	0.23% (0.18)	4.7	EUR	IBS		
	1.11% (0.07)	0.25% (0.20)	4.4	EUR	TSI		
	0.08% (0.02)	0.04% (0.07)	2.0	AFR	LWK		
Sankararaman *et al.*, 2016*							
Conditional Random Field					*SGDP*	Altai	[2]
	1.37% (0.11)	0.26% (0.18)	5.3	AMR	*		
	1.40% (0.12)	0.23% (0.18)	6.1	CAS	*		
	1.39% (0.11)	0.32% (0.28)	4.3	EAS	*		
	1.54% (0.12)	0.42% (0.36)	3.7	OCE	*		
	1.19% (0.11)	0.40% (0.26)	3.0	SAS	*		
	1.06% (0.12)	0.18% (0.19)	5.9	EUR	*		
Sankararaman *et al.*, 2016*							
Conditional Random Field					*SGDP*	Denisovan	[2]
	0.05% (0.01)	0.00% (0.00)	-	AMR	*		
	0.05% (0.01)	0.00% (0.00)	-	CAS	*		
	0.06% (0.02)	0.00% (0.01)	-	EAS	*		
	0.85% (0.43)	0.18% (0.17)	4.7	OCE	*		
	0.06% (0.03)	0.01% (0.03)	6.0	SAS	*		
	0.02% (0.01)	0.00% (0.00)	-	EUR	*		
DICAL-ADMIX							
HMM with demographic model					*1k Genomes*	Altai	[3]
	1.48%	0.38%	3.9	EUR	CEU		
	1.80%	0.54%	3.3	EAS	CHB+CHS		
ARGWeaver-D							
Bayesian ARG inference					*SGDP*	both Vindija	
and Altai	[4]						
	1.9%	0.25%	7.6	EUR	Basque		
	2.5%	0.50%	5.0	OCE	Papuan		
	0.25%	0.20%	1.3	AFR	San		
	0.25%	0.25%	1.0	AFR	Mandenka		
`hmmix`							
reference-free HMM					*1k Genomes*	no reference	[5]

	2.16% (0.09)	0.41% (0.21)	7.0	EUR	FIN		
	2.06% (0.08)	0.44% (0.21)	6.4	EUR	CEU		
	2.06% (0.08)	0.43% (0.18)	6.4	EUR	GBR		
	1.97% (0.10)	0.45% (0.23)	6.2	AMR	PUR		
	2.08% (0.09)	0.44% (0.21)	6.2	AMR	CLM		
	2.01% (0.08)	0.42% (0.18)	6.1	EUR	IBS		
	2.20% (0.10)	0.49% (0.21)	5.8	AMR	MXL		
	2.31% (0.12)	0.52% (0.22)	5.3	AMR	PEL		
	2.01% (0.08)	0.46% (0.19)	5.2	EUR	TSI		
	2.30% (0.12)	0.62% (0.28)	4.8	SAS	PJL		
	2.60% (0.09)	0.70% (0.30)	4.7	EAS	CHS		
	2.32% (0.09)	0.61% (0.25)	4.6	SAS	GIH		
	2.36% (0.08)	0.69% (0.30)	4.4	SAS	ITU		
	2.58% (0.09)	0.70% (0.27)	4.3	EAS	JPT		
	2.37% (0.08)	0.68% (0.26)	4.2	SAS	STU		
	2.56% (0.08)	0.71% (0.25)	4.2	EAS	KHV		
	2.58% (0.08)	0.77% (0.31)	4.2	EAS	CDX		
	2.61% (0.08)	0.74% (0.27)	4.1	EAS	CHB		
	2.41% (0.08)	0.74% (0.29)	3.8	SAS	BEB		
`hmmix`**							
reference-free HMM					*SGDP*	no reference	[5]
	2.56% (0.14)	0.40% (0.27)	8.9	CAS	**		
	1.94% (0.09)	0.36% (0.22)	8.2	EUR	**		
	2.65% (0.13)	0.51% (0.25)	6.3	EAS	**		
	2.30% (0.12)	0.46% (0.29)	5.9	SAS	**		

Table S1. Genome-wide archaic coverage levels on autosomes and chromosome X (chrX) inferred from human genomic data. Coverage is the per-base pair proportion of the genome inferred to have come from archaic sources, found either on the combined 22 autosomes, or on chromosome X. The standard deviation across individuals is in parentheses. The fourth column shows the ratio of autosomal archaic coverage to chromosome X archaic coverage (Aut:ChrX), which is always at least equal, and can range up to 18.8. All groups outside of Continental Africa have at least 3.3 times more relative coverage on autosomes than chromosome X.

See Table 1 for continental group and 1kG sample group abbreviations. Altai Neanderthal published in Prüfer *et al.*, 2014 [6]; Vindija Neanderthal published in Prüfer *et al.*2017 [7]; Denisovan published in Meyer *et al.*, 2012 [8]. Inference methods were applied to various data sources: 1k Genomes [9], and SGDP [10]. * See Supplementary Table 2 of Sankararaman *et al.*, 2016 [2] for group-specific coverage estimates. ** See Supplementary Dataset S5 of Skov *et al.*, 2018 [5] for group-specific coverage estimates.

References

1. Sankararaman S, Mallick S, Dannemann M, Prüfer K, Kelso J, Pääbo S, et al. The genomic landscape of Neanderthal ancestry in present-day humans. Nature. 2014;507(7492):354–357. doi:10.1038/nature12961.

2. Sankararaman S, Mallick S, Patterson N, Reich D. The Combined Landscape of Denisovan and Neanderthal Ancestry in Present-Day Humans. Current Biology. 2016;26(9):1241–1247. doi:10.1016/j.cub.2016.03.037.

3. Steinrücken M, Spence JP, Kamm JA, Wieczorek E, Song YS. Model-based detection and analysis of introgressed Neanderthal ancestry in modern humans. Molecular Ecology. 2018;27(19):3873–3888. doi:10.1111/mec.14565.

4. Hubisz MJ, Williams AL, Siepel A. Mapping gene flow between ancient hominins through demography-aware inference of the ancestral recombination graph. PLOS Genetics. 2020;16(8):e1008895. doi:10.1371/journal.pgen.1008895.

5. Skov L, Hui R, Shchur V, Hobolth A, Scally A, Schierup MH, et al. Detecting archaic introgression using an unadmixed outgroup. PLoS Genetics. 2018;14(9):e1007641. doi:10.1371/journal.pgen.1007641.

6. Prüfer K, Racimo F, Patterson N, Jay F, Sankararaman S, Sawyer S, et al. The complete genome sequence of a Neanderthal from the Altai Mountains. Nature. 2014;505(7481):43–49. doi:10.1038/nature12886.

7. Prüfer K, de Filippo C, Grote S, Mafessoni F, Korlević P, Hajdinjak M, et al. A high-coverage Neandertal genome from Vindija Cave in Croatia. Science (New York, NY). 2017;358(6363):655–658. doi:10.1126/science.aao1887.

8. Meyer M, Kircher M, Gansauge MT, Li H, Racimo F, Mallick S, et al. A High-Coverage Genome Sequence from an Archaic Denisovan Individual. Science. 2012;338(6104):222–226. doi:10.1126/science.1224344.

9. The 1000 Genomes Project Consortium. A global reference for human genetic variation. Nature. 2015;526(7571):68–74. doi:10.1038/nature15393.

10. Mallick S, Li H, Lipson M, Mathieson I, Gymrek M, Racimo F, et al. The Simons Genome Diversity Project: 300 genomes from 142 diverse populations. Nature. 2016;538(7624):201–206. doi:10.1038/nature18964.
