# Supplementary material for: Integrating sex-bias into studies of archaic introgression on chromosome X
Source: PLoS Genet. 2023 Aug 14;19(8):e1010399. doi: 10.1371/journal.pgen.1010399 (PMC10449224; doi:10.1371/journal.pgen.1010399)
Supplement: S2 Table — Coverage estimates in extant human groups are presented in Fig 1. (PDF) [file pgen.1010399.s010.pdf]

Table S1. Methods used to estimate archaic coverage on chromosome X. Coverage estimates in extant human groups are presented in Fig. 1 and S2 Table.

Fig 1 label	Estimation method	Data Source*	Archaic specificity
ARGWeaver-D	Hubisz *et al.*, 2020 [1]	SGDP	Neanderthal
Dical-admix	Steinrücken *et al.*, 2018 [2]	1kG	Neanderthal
Sank14	Sankararaman *et al.*, 2014 [3]	1kG	Neanderthal
Sank16Neand	Sankararaman *et al.*, 2016 [4]	SGDP	Neanderthal
Sank16Denis	Sankararaman *et al.*, 2016 [4]	SGDP	Denisovan
`hmmix`1kG	`hmmix` [5]	1kG	none
`hmmix`SGDP	`hmmix` [5]	SGDP	none

*SGDP refers to Simons Genome Diversity Project [6]; 1kG refers to the 1000 Genomes Project [7].

References

1. Hubisz MJ, Williams AL, Siepel A. Mapping gene flow between ancient hominins through demography-aware inference of the ancestral recombination graph. PLOS Genetics. 2020;16(8):e1008895. doi:10.1371/journal.pgen.1008895.

2. Steinrücken M, Spence JP, Kamm JA, Wieczorek E, Song YS. Model-based detection and analysis of introgressed Neanderthal ancestry in modern humans. Molecular Ecology. 2018;27(19):3873–3888. doi:10.1111/mec.14565.

3. Sankararaman S, Mallick S, Dannemann M, Prüfer K, Kelso J, Pääbo S, et al. The genomic landscape of Neanderthal ancestry in present-day humans. Nature. 2014;507(7492):354–357. doi:10.1038/nature12961.

4. Sankararaman S, Mallick S, Patterson N, Reich D. The Combined Landscape of Denisovan and Neanderthal Ancestry in Present-Day Humans. Current Biology. 2016;26(9):1241–1247. doi:10.1016/j.cub.2016.03.037.

5. Skov L, Hui R, Shchur V, Hobolth A, Scally A, Schierup MH, et al. Detecting archaic introgression using an unadmixed outgroup. PLoS Genetics. 2018;14(9):e1007641. doi:10.1371/journal.pgen.1007641.

6. Mallick S, Li H, Lipson M, Mathieson I, Gymrek M, Racimo F, et al. The Simons Genome Diversity Project: 300 genomes from 142 diverse populations. Nature. 2016;538(7624):201–206. doi:10.1038/nature18964.

7. The 1000 Genomes Project Consortium. A global reference for human genetic variation. Nature. 2015;526(7571):68–74. doi:10.1038/nature15393.
