# Supplementary material for: Integrating sex-bias into studies of archaic introgression on chromosome X
Source: PLoS Genet. 2023 Aug 14;19(8):e1010399. doi: 10.1371/journal.pgen.1010399 (PMC10449224; doi:10.1371/journal.pgen.1010399)
Supplement: S1 Appendix — (PDF) [file pgen.1010399.s011.pdf]

S1 Appendix

We performed additional simulations with chromosome-specific exon and recombination maps. Simulations of realistic chromosome architecture used different exon and recombination rate maps for simulated autosomal and chromosome X chromosomes. These simulations modeled a randomly-selected 50Mb portion of the long arm of either chromosome 7 (`chr7:101351049-151351049`) or chromosome X (`chrX:101351049-151351049`). Bulk properties of these regions are presented in Table A1.

Exon locations were defined as the protein coding regions in the basic gene annotation map obtained from GENCODE Release 37 (GRCh37; `https://www.gencodegenes.org/human/release_37lift37.html`) [1].

Recombination rate maps were obtained from Bhérer *et al.*, 2017 [2], and reflect data from individuals with European ancestry. We used a sex-averaged map for chromosome 7 simulations.

**Inheritance model**	**Avg. Exon Density**	**Avg. Recombination Rate (cM/bp)**
Autosomal	0.026	1.3×10^{-7}
Chromosome X	0.019	2.2×10^{-7}

Table A1. Properties of simulated regions of realistic human chromosome architecture.

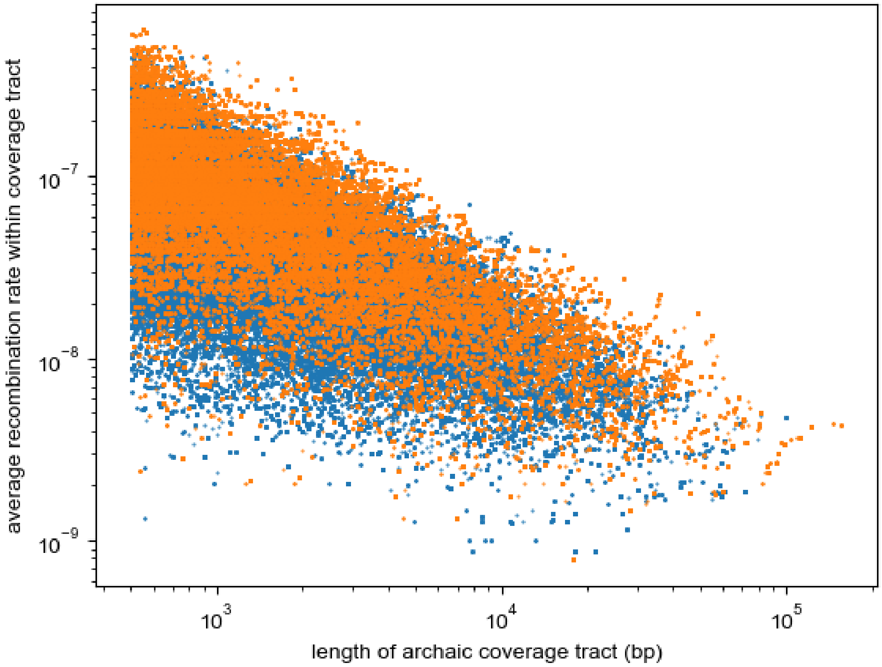

Fig A1. Recombination rate and length of archaic coverage tract are negatively correlated. Identical to S4 Fig. B, plotted with data from chromosome-specific exon and recombination rate maps as described in Table A1.

References

1. Frankish A, Diekhans M, Ferreira AM, Johnson R, Jungreis I, Loveland J, et al. GENCODE reference annotation for the human and mouse genomes. Nucleic Acids Research. 2019;47(D1):D766–D773. doi:10.1093/nar/gky955.

2. Bhérer C, Campbell CL, Auton A. Refined genetic maps reveal sexual dimorphism in human meiotic recombination at multiple scales. Nature Communications. 2017;8. doi:10.1038/ncomms14994.
