# Supplementary material for: Integrating sex-bias into studies of archaic introgression on chromosome X
Source: PLoS Genet. 2023 Aug 14;19(8):e1010399. doi: 10.1371/journal.pgen.1010399 (PMC10449224; doi:10.1371/journal.pgen.1010399)
Supplement: Attachment — Submitted filename: chevyetal_response.pdf [file pgen.1010399.s012.pdf]

**Reviewer #1**

**Summary**

In this manuscript, the authors explore the dearth of Neanderthal and Denisovan DNA on the X chromosome, compared to the autosomes. This pattern has been observed in several studies of archaic ancestry in modern humans, and the main point of this study is analysing how sex-biased introgression may have influenced this, by using simulations. This is a great example of using existing datasets and simulations for novel insights, and a manuscript where things are described with clarity and in a comprehensive manner. I have only minor points which I suggest to address.

We thank the reviewer for the close reading of our manuscript, and for their positive assessment of the submitted draft. Below address their minor comments.

1) The first point concerns limitations of the methods to infer introgressed fragments. Such methods depend on mutation density – as a consequence, regions with low mutation rates will be more difficult to be identified as introgressed, even if they are. This is not a limitation of this study per se, rather of the underlying data for present-day humans in the initial observations. As I understood, heterogeneous mutation rates were not applied in the simulations (the link to the GitHub repo does not work yet), but since the "true" fragments are known, it does not matter for the result. However, this may be a factor in the real data leading to the hypothesis, because if the mutation rate of the X chromosome is lower, which I believe is the case, introgressed fragments would be less likely to be discovered. Most likely, it does not explain the whole pattern, but it might be useful to discuss this.

   We thank the reviewer for this important point. First, we note that the GitHub repository (https://github.com/ramachandran-lab/archaic-chrX-sexbias) is now public and apologize it was not previously.

   The mutation rate difference between the autosomes and chromosome X complicates accurately calling archaic ancestry in empirical datasets. To address this concern in this revised manuscript, we have re-run the introgression segment calling method hmmix (Skov, L., Hui, R., Shchur, V., Hobolth, A., Scally, A., Schierup, M. H., & Durbin, R. (2018). Detecting archaic introgression using an unadmixed outgroup. *PLoS Genetics*, *14*(9), e1007641. https://doi.org/10.1371/journal.pgen.1007641) on the 1kG data underlying Figure 2, modifying the relative autosomal and chromosome X emission parameters to reflect the lower mutation rate on chromosome X. This change brings our aut:chrX results in the 1kG data more in line with those seen in our simulations.

2) I found it a bit surprising that the absence of both archaic mitochondrial DNA and Y chromosomes in modern humans, with a likely replacement of both in Neanderthals (10.1126/science.abb6460, 10.1038/ncomms16046), was not mentioned in this context. Sex-biased migration, for example, with a male bias, would have increased the chances

of transmission of the Y chromosome, but we don’t observe it.

We thank the reviewer for pointing out these two references proposing that both mitochondrial DNA and Y chromosome in archaic humans were replaced by the modern human versions. Analyzing all markers (in mitochondria, Y chromosome, autosomes and X chromosome) will lead to interesting insights about the introgression events from modern humans into archaic humans and introgression events from archaic humans into modern humans. Since in our study we do not simulate introgression from modern humans into Neanderthals for these two non-recombining loci (mtDNA and Y chromosome), we only comment in the discussion. Specifically we added the following:

In addition, we have only focused on contrasting patterns of archaic introgression in the autosomes and the X chromosome. However, studies of non-recombining loci like the Y chromosome and the mitochondria have proposed that both the mitochondria and Y chromosome may have been replaced in late Neanderthals after an introgression from early modern humans into Neanderthals (Petr et al., 2020; Posth et al., 2017). Interestingly, in this scenario, no sex chromosome/autosomal differences are observed (Hubisz et al., 2020). Modeling all chromosomes might provide insights into how the interplay of natural selection, sex-bias and different inheritance patterns result in distinct patterns of introgression.

3) On the other hand, gene flow from humans into Neanderthals seems to have been similar on the X chromosome and the autosomes (using ARGweaver, see citation [3] in the manuscript). This could be used to argue in favour of a sex-biased migration in the archaic-to-human scenario, however with a caveat of likely lower efficacy of selection in Neanderthals than modern humans (10.1073/pnas.1405138111).

   We thank the reviewer for alerting us to this finding. We added a small comment (see response to previous point (#2) by the reviewer).

4) The authors mention that introgression is common in other species (line 276). It might actually be useful to discuss what has been observed in the few species where this question was addressed: chimpanzees show a depletion of bonobo ancestry (measured with D-statistics; 10.1126/science.aag2602); bonobos show a depletion of “ghost” ancestry (8-fold compared to autosomes, using the Skov method; 10.1038/s41559-019-0919-x); the X chromosome seems not to differ from autosomes in introgression proportions in Southeast Asian macaques (10.1098/rspb.2021.1756); high-altitude pigs carry a long, positively selected haplotype that likely introgressed from an extinct species (10.1038/ng.3199). This kind of question is not completely

unexplored in other species - especially our closest living relatives, which may serve as a direct comparison.

We have added these references in our discussion. The text now reads:

Finally, we only consider parameters related to modern humans, but several studies have shown that introgression is common in other species (Ai et al., 2015; de Manuel et al., 2016; Evans et al., 2021; Kuhlwilm et al., 2019). For example, introgression from bonobos into the ancestors of central and eastern chimpanzees has been detected (de Manuel et al., 2016), and like humans, the amount of introgression is smaller on the X chromosomes. Another study detected introgression from a ghost lineage into bonobos, and introgression was also depleted on the X chromosome (Kuhlwilm et al., 2019). Our model structure can be adapted to other organisms to provide insights into speciation and how demography, genomic structure, sex-bias and natural selection have shaped the distribution of introgressed variants on the autosomes and chromosome X.

5) Somewhat related to the previous point, this might be added as a caveat: indeed sex-biased introgression might have contributed to what we see in humans, but given such similarities on the X chromosome of our closest living relatives (chimpanzees and bonobos), with very different sex-biased migration patterns and behaviours of their present-day populations, it is still an option that deleterious mutations are causing this. There is uncertainty of their past and our own past migration patterns, and the authors show that any such effect would have been immediate (Fig. 5) - so it seems very unlikely that this could be fully resolved. Here, I want to emphasize that this doesn't impact the merit of the study, which lies in demonstrating the reasonable possibility of this factor. So, this last point is rather to think about the biology, and probably doesn't need to be concretely addressed in the manuscript.

We certainly agree with the reviewer that purging of deleterious load from chromosome X is a major driver of the pattern of less archaic introgression on chromosome X than autosomes– whether through Dobzhansky Muller interactions, a faster-X response to background selection, or other evolutionary processes. In fact, our simulations all implement a "genetic load hypothesis" (Harris, K., & Nielsen, R. (2016). The Genetic Cost of Neanderthal Introgression. *Genetics*, *203*(2), 881–891. https://doi.org/10.1534/genetics.116.186890) scenario where archaic segments are deleterious in the modern human background solely due to containing weakly deleterious mutations that were not purged in the archaic population due to its smaller effective population size.

The timescale of purging due to sex-bias is indeed very short, as demonstrated both in our simulations (Figs. 3, 4) and in theory (Goldberg, A., & Rosenberg, N. A. (2015). Beyond 2/3 and 1/3: The Complex Signatures of Sex-Biased Admixture on the X Chromosome. *Genetics*, *201*(1), 263–279.

https://doi.org/10.1534/genetics.115.178509). We have added some additional text discussing Fig. 4 in response to this point (lines 170-186).

---

**Reviewer #2**

**Summary**

In this manuscript the authors are studying the remains of the admixture event between humans and Neanderthals. While the autosomes retain around 2% of Neanderthal ancestry the X chromosome has been depleted of archaic ancestry. The authors find that there is around 7.9 times more archaic sequence on the autosomes compared to the X chromosomes when studying individuals from the 1000 genomes project. They find differences between different human groups e.g. Americas has less archaic ancestry on the X chromosome compared to South Asian samples.

What is novel is that the authors look into the effect of sex-bias. They use simulations to argue that the results can be modeled by sex-bias (more neanderthal men than women). The manuscript is clearly written and its easy to follow.

We thank reviewer 2 (Laurits Skov) for the detailed review and constructive comments, and for appreciating the contribution of this manuscript to the literature on archaic introgression in the human genome. We note that, in rerunning our analyses based on reviewer 2's comments (particularly in our application of HMMix, (Skov, L., Hui, R., Shchur, V., Hobolth, A., Scally, A., Schierup, M. H., & Durbin, R. (2018). Detecting archaic introgression using an unadmixed outgroup. *PLoS Genetics*, *14*(9), e1007641. https://doi.org/10.1371/journal.pgen.1007641), our estimates of autosomal archaic tract lengths have changed. We discuss this further below in response to comment 1.2, as well as our other point-by-point responses to this helpful review.

Major comments

1.1) The authors find a Auto:X ratio of around 7.9 in the 1000 genomes data but their simulations only show a Auto:X ratio of 3 (Figure 3A,B). So there seems there are additional things at play which have much larger effects than the sex-bias.

The reduction in archaic ancestry on the X chromosome is a combination of:

dominance model (recessive or additive) + sex-bias + selective sweep + differences in recombination map + Hybrid incompatibility loci + Background selections + Demographics changes specific to different population

We appreciate the focus on sex-bias but in light of a recent preprint it would be good to pay extra attention to selective sweeps.

A recent preprint (Skov et al.)(Extraordinary selection on the human X chromosome associated with archaic admixture | bioRxiv) found that there are 17 Mb of the X

chromosomes in non-africans are affected by selective sweeps. These sweeps make up 10% of the X chromosome and effected haplotypes have almost no archaic admixture (mean = 0.0045%). The regions can be found in in Supplementary table S2 (start and end are in 100kb windows so a start of 195 means 19,500,000 bp).

This would presumably have an impact on the Auto:X ratio and make it appear higher than it actually is in 1000genomes data for instance. It would be good to repeat the analysis of the 1000 genomes data but excluding these regions.

We thank the reviewer for this suggestion and agree that reconciling this difference is important. We have now rerun HMMix to call archaic tracts in the 1kG data again for both the autosomes and chromosome X and updated Figure 2 as well as our discussion of it (lines 76-89).

Below we show the previously submitted version of Figure 2 (left) and the revised version (right). The revised version is run with modified chromosome X emissions parameters (see comment 1.2 below), and all 75% regions associated with selective sweeps have been removed using coordinates from Table S2 of Skov, L., Macià, M. C., Lucotte, E., Cavassim, M. I. A., Castellano, D., Schierup, M. H., & Munch, K. (2022). *Extraordinary selection on the human X chromosome associated with archaic admixture* (p. 2022.09.19.508556). bioRxiv. https://doi.org/10.1101/2022.09.19.508556.

As the reviewer predicted, the observed ratios are lower and more in line with what is observed in simulations and what has previously been discussed in empirical analyses. We also note that the per-base-pair autosomal coverage has decreased by roughly a factor of two. We presume that the original tracts were those found on either autosomal haplotype, not in diploid mode as we originally interpreted. Overall Figure 2 now underscores even more deeply that the components of the autosomal to X chromosomal ratio of archaic ancestry are important to study to understand the legacy of archaic introgression in diverse human genomes.

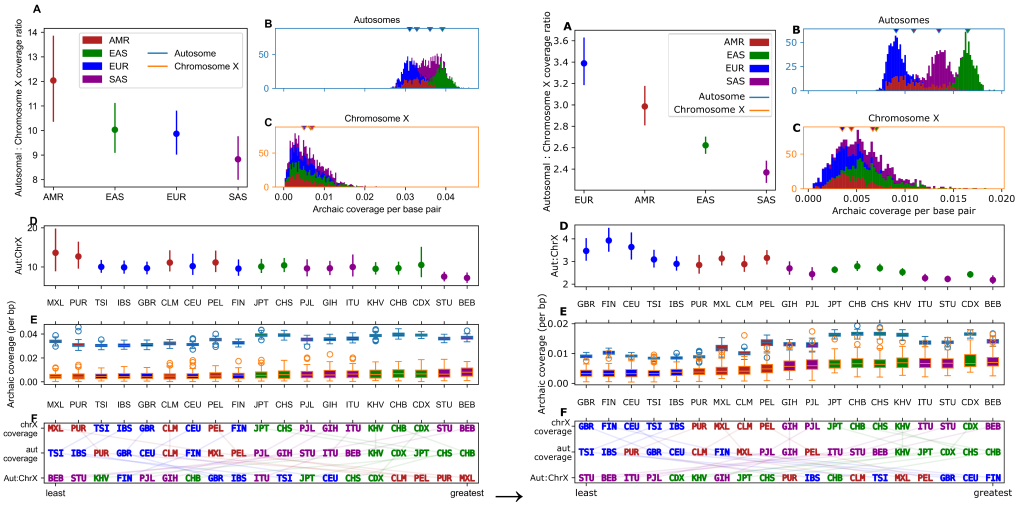

1.2) Another issue is that when using hmmix (Skov et al 2018) one needs to change the parameters for the X chromosome. Since ¾ of mutations are passed on from men (Jónsson et al 2017) but the X chromosome only spends 1/3 of its time in men the mutation rate will be lower compared to the autosomes. It turns out that the emission parameters of hmmix needs to be multiplied by 5/6 on the X. This also changes the ratio for different 1000 genomes populations – see Supplementary table S3 for how it changes the ratios in the SGDP dataset (Skov et al.). We have also attached the relevant table with the calculated Auto:X ratios.

We appreciate this point from the reviewer; initially we had used tracts called by Laurits Skov released to us via personal communication. We are thankful he told us that he had not run HMMix with X-specific emission parameters for the 1kG data, and so we reran these and have updated Figs. 1, 2, and Table S1. We discuss these new results in lines 76-89.

We feel that taking questions 1.1 and 1.2 into account will likely lower the Auto:X ratio to a point that is more compatible with the simulation studies – which would allow for some more concrete inferences about the human/neanderthal meeting.

As reviewer 2 predicted, we now observe empirical ratios similar to the simulation studies.

1.3) The authors spend a lot of time discussing the difference in fragment length distribution of archaic segments on the X chromosome and the autosomes. (Figure 4) But as far as we can tell the results are not discussed and compared with real data – could the authors elaborate a bit on what we can learn from this analysis when comparing it to the 1000 genomes data? Also, It is not clear which new insights can be gained from studying the admixture proportion over time (Figure 5) that we didn't already know from references (Harris and Nielen 2016, Kim et al 2018, Petr et al 2019) - perhaps the authors could elaborate a bit here?

Because this manuscript focuses on the potential effects of sex-biased introgression, rather than sex chromosome inheritance generally, we have reduced the discussion of archaic segment lengths in the main text, and moved the tract length figure (formerly Fig. 4) to the supplement as Fig. S4. It now sits alongside Fig. S5, which also communicates that chromosome X archaic tracts are generally longer than autosomal tracts. While we agree with the reviewer that analyzing the archaic segment lengths in real data would be interesting, we do not have the power to infer sex-bias from these data alone, and would only be able to discuss general patterns of chromosome X vs. autosomal recombination and inheritance.

We have expanded the discussion of the time course figure (formerly Fig. 5, now Fig. 4) to more directly address the effects of sex-bias, which was not explored in previous studies of the time course of archaic coverage purging. Please see lines 179-187.

1.4) We really liked Supplementary figure 2! Even more than the current main Figure 3. Maybe Supplementary figure 2 should be used in the main text instead of Figure 3 because it is really helpful to have the neutral scenario along side the scenarios where they simulated sex-bias and dominance model. It would also be helpful to see the neutral curve in Supplementary figure 2 simulated from the scenario described (Supplementary figure 1) instead of having the theoretical distribution. Also there is something strange going on with the confidence intervals - why is the ratio for the additive effect higher for a female fraction if 0.4 compared to 0.2 – it is strange that the confidence intervals are so narrow.

We thank the reviewer for the positive feedback about Fig. S2! We also think the theoretical curve is interesting. We present the relevant part of the theoretical curve alongside the simulated aut:chrX ratios in Fig. 3A as horizontal chartreuse line segments. In response to this comment we have run some simulations of a “neutral” scenario. Results are provided in a new supplemental figure (Fig S8). The mean aut:chrX ratios in these simulations somewhat underestimate the theoretical expectations. We anticipate that the non-monotonicity of the data in Fig. S2 is due to the demographic stochasticity demonstrated in Fig. S8A.

Minor comments

2.1) Fig 1. Would it be possible to add text to the diagonal lines indicating what the ratio is. For instance: Auto:X=1, Auto:X=2 and so on it would improve the readability of the figure. Perhaps you could also omit plotting the mean 7.9 as it is on top of the ratio=8 line (also because that ratio might be too high see 1.2).

Figure 1 has been updated with labels for the Aut:chrX clines.

2.2) Would it make more sense to have exchange Table 1 in the main text for SI Table 2 – this table have the Auto:X estimates which would be helpful to have in the main text instead of having to look it up in the supplement. You could still keep the population abbreviations of course.

Table S1 contains the Aut:chrX ratio estimates calculated from published results of introgression-detection methods. Note that we have updated the table to reflect introgression calls from our re-running of hmmix on the 1kG data, as well as the results on SGDP data from Skov, L., Macià, M. C., Lucotte, E., Cavassim, M. I. A., Castellano, D., Schierup, M. H., & Munch, K. (2022). *Extraordinary selection on the human X chromosome associated with archaic admixture* (p. 2022.09.19.508556). bioRxiv. https://doi.org/10.1101/2022.09.19.508556

We have plotted each line of Table S1 as a point in Fig.1 to summarize the large

amount of information in the table.  We hope that the color and symbol keys can help readers translate particular results of interest between Table S1 and Fig. 1.

2.3) In line 175 the authors state that:

"Panels A and B reflect data from simulations without introgressor sex-bias; panel C illustrates that regardless of introgressor sex-bias, the 95th percentile of archaic coverage tract lengths on chromosome X is longer than the 95th percentile of tract lengths on an autosome"

Shouldn't this be panel D – as there is no sex-bias in panel C.

Yes, thanks for pointing out this typo – the panel label was incorrect in the text.  We have since moved this figure to the supplement as Fig. S4.

2.4) We would aviod using language such as "Differences among the coverage ratios are not cleanly correlated". We would reserve words such as "significant" and "correlation" for when you are performing statistical tests. You could write Differences among the coverage ratios are not sorted by popultion". In addition in Figure 4, panel A the Y-axis is labeled as frequency but it should be counts.

Line 79 now reads: "The order of continental groups by coverage ratio does not match their order by absolute amounts of coverage".

Figure 4A's y-axis label has been updated to read "counts", and is now located in the supplement as Fig. S4.

2.5) In line 75 you introduce the term aut:chrX but it is not defined. Could you add in in line 56 when you write: The autosomal to chromosome X coverage ratio (aut:chrX)

Line 56 now reads: "The autosomal to chromosome X coverage ratio (aut:chrX) reflects how many times[...]".

2.6) We are puzzled by Figure 5, Panel A. Why are does the archaic coverage change for the autosomes when you change the proportion of male introgressers – wouldn't this always be the same?

We also expect the archaic coverage rate on the autosomes to be independent of the proportion of male introgressors.  We observe no systematic differences between the introgressor sex-ratios (colors) in panel A ('Autosome') of the timecourse figure (formerly Fig.5, now Fig. 4).  After changes to the text addressing point 1.3, only chromosome X is discussed in the context of sex-bias in the results.
