# Supplementary material for: Integrating sex-bias into studies of archaic introgression on chromosome X
Source: PLoS Genet. 2023 Aug 14;19(8):e1010399. doi: 10.1371/journal.pgen.1010399 (PMC10449224; doi:10.1371/journal.pgen.1010399)
Supplement: Attachment — Submitted filename: reviewer_response_rev2.pdf [file pgen.1010399.s013.pdf]

Reviewer #1

The authors have fully addressed the minor points from my review. In my opinion, together with the improvements in response to reviewer 2, this is a great manuscript for publication in Plos Genetics.
Of note and related to my comment 4, meanwhile there is another study on archaic introgression in a close relative of humans, gorillas (10.1101/2022.12.19.521012). There, we also find a depletion of introgression on the X chromosome, so it seems indeed quite common.

Thank you for your kind words and thoughtful reviews.

Reviewer #2

The authors have responded to all my comments and I am happy to say that the manuscript has improved greatly - good job!
The simulation results are now much more similar to what is observed in 1000 genomes data. I think the simulation part of the study is solid so I only have major concern left.

When looking at Figure 2 (or Table 2 for that matter) It seems that Europeans have around 0.7% archaic coverage per base and East Asians have around 1.6% archaic coverage per base which is twice as much. For previous studies this difference is not as great (Skov et al 2018 finds 1.94% in EUR and you now find 0.91 in CEU for instance - I found the numbers in your Table 2) so I suspect something went wrong when running hmmix. If I was to take a guess I would say that you might be using individuals in your outgroup that have West Eurasian ancestry such as the 1000 genomes populations of LWK and GWD (see Figure 2 in https://www.nature.com/articles/nature15393). Which individuals did the authors include in the outgroup when running hmmix?

Once this issue is addressed I will have no further comments.

Thank you for bringing this issue to our attention. We had indeed used LWK and GWD individuals in the outgroup.
After removing those individuals from the outgroup and rerunning hmmix we saw unreasonably high chrX coverage estimates. We then further modified our process to remove all chrX inferred coverage tracts with no derived alleles from several high coverage archaic reference genomes. These changes are described in Methods.

We have also excluded LWK and GWD individuals from the supervised AFR group in our chrX ADMIXTURE runs underlying Figure S7. This change resolves a clearer pattern of admixture in AMR populations.
